# Multiple Determinants of Whole and Regional Brain Volume among Terrestrial Carnivorans

**DOI:** 10.1371/journal.pone.0038447

**Published:** 2012-06-13

**Authors:** Eli M. Swanson, Kay E. Holekamp, Barbara L. Lundrigan, Bradley M. Arsznov, Sharleen T. Sakai

**Affiliations:** 1 Department of Zoology, Michigan State University, East Lansing, Michigan, United States of America; 2 BEACON Center for the Study of Evolution in Action, Michigan State University, East Lansing, Michigan, United States of America; 3 Michigan State University Museum, Michigan State University, East Lansing, Michigan, United States of America; 4 Neuroscience Program, Michigan State University, East Lansing, Michigan, United States of America; 5 Department of Psychology, Michigan State University, East Lansing, Michigan, United States of America; University of Lethbridge, Canada

## Abstract

Mammalian brain volumes vary considerably, even after controlling for body size. Although several hypotheses have been proposed to explain this variation, most research in mammals on the evolution of encephalization has focused on primates, leaving the generality of these explanations uncertain. Furthermore, much research still addresses only one hypothesis at a time, despite the demonstrated importance of considering multiple factors simultaneously. We used phylogenetic comparative methods to investigate simultaneously the importance of several factors previously hypothesized to be important in neural evolution among mammalian carnivores, including social complexity, forelimb use, home range size, diet, life history, phylogeny, and recent evolutionary changes in body size. We also tested hypotheses suggesting roles for these variables in determining the relative volume of four brain regions measured using computed tomography. Our data suggest that, in contrast to brain size in primates, carnivoran brain size may lag behind body size over evolutionary time. Moreover, carnivore species that primarily consume vertebrates have the largest brains. Although we found no support for a role of social complexity in overall encephalization, relative cerebrum volume correlated positively with sociality. Finally, our results support negative relationships among different brain regions after accounting for overall endocranial volume, suggesting that increased size of one brain regions is often accompanied by reduced size in other regions rather than overall brain expansion.

## Introduction

The considerable brain size variation evident among mammals is thought to result primarily from variation in body size [1]–[3] and secondarily from variation in encephalization, which involves changes in brain size independent of body size [1]. Although body size often explains as much as 95% of the variance in absolute brain size, brain sizes at a given body size can nevertheless range over an order of magnitude [4], and a number of different factors have been proposed to explain this variation. Some of the most prominent factors proposed to explain variation in encephalization include social complexity [5], [6], life history [7], recent evolutionary changes in body size [8], and complexity in the non-social environment as indicated by such variables as home range size, manual dexterity required during food processing, and factors related to diet such as complexity of foraging behavior [9], [10].

The ‘social brain hypothesis’, which argues that degree of encephalization increases with the complexity of the intraspecific social environment [6], [11]–[13], is one of the most popular hypotheses proposed to explain variation in encephalization. This hypothesis is strongly supported by data gathered from primates (see [14]). However, its generality among non-primate mammals is poorly understood, as it has only been tested in a few taxa [15], [16], and different studies have yielded conflicting results even when such results were based on the same data (e.g. compare [17], [18]). While the social brain hypothesis is probably the most widely studied explanation for encephalization, life history traits have also been suggested to influence encephalization. Gestation length, for example, has been linked to degree of encephalization because, relative to the rate of body size growth, prenatal brain growth is far more rapid than postnatal brain growth [1], [19], [20]. Prolonged lactation has also been suggested to result in increased brain size, as the nutritional benefits of extended access to milk may often be required to help offset the high metabolic cost of neural tissue [21]–[23]. Finally, longevity has been proposed to increase degree of encephalization for adaptive reasons; specifically, species with larger brains may be able to respond better to environmental changes requiring resource shifts during an extended lifespan [24], [25]. In addition to social complexity and life history, characteristics of the physical environment might influence brain size independent of body size. For example, home range size has been suggested to relate to brain size because larger home ranges require species to utilize complex information about food location and distribution that would not be necessary for species more constantly in contact with their food sources [9]. Use of the forelimb in food processing has been suggested to relate to brain size [26], reflecting the link between manual dexterity and motor or somatosensory cortex [27]–[29]. Finally, diet may relate to degree of encephalization either as an energetic constraint due to the metabolic ‘expense’ of brain tissue [30], [31], or because some diets require more complex foraging or processing techniques [9], [32]. Despite the fact that these hypotheses relating to sociality, the non-social environment, and life history are generally viewed in a competitive framework, it is highly likely that more than one of these factors operate in a given species to shape brain volume [33], [34]. Although some research has considered multiple factors (e.g. [35]–[37]), it is still common to examine only one of these potential sources of variation, despite the demonstrated importance of considering multiple hypotheses simultaneously [38].

Most of the hypotheses purporting to explain encephalization generalize specific functions to the entire brain, yet, different brain functions are often associated with neural activity in different areas of the brain [39]. Thus, many hypotheses proposed to explain overall encephalization should perhaps preferentially be applied to specific brain regions (e.g. [40]–[42]). This is rarely done, likely due in part to the difficulty of identifying and separating brain regions in a large set of taxa. Moreover, there is considerable controversy regarding the extent to which different brain regions can evolve independently. Expansion of particular brain regions may be the result of concerted change due to developmental linkages among brain regions [43]. Conversely, there is evidence that brain regions evolve independently, known as ‘mosaic evolution’ [44]. Both processes undoubtedly play roles in brain evolution ([45], pgs. 157–159), but their relative importance critically affects our ability to recognize adaptive variation in specific brain regions; we may be able to identify such adaptive variation in brain regions only if mosaic evolution is common. An additional consideration is simply that the brain must fit within the skull ([45], pg. 131). This simple requirement means that if antagonistic selection or developmental factors constrain skull size evolution, then increases in one brain region must be accompanied by concomitant decreases in other regions rather than overall increases in encephalization (e.g. [40], [42]).

In addition to these adaptive explanations for variation in encephalization, other hypotheses have been proposed that are not directly adaptive. For example, some variation in the degree of encephalization has commonly been hypothesized to arise from an evolutionary ‘lag’, where body size evolves first, later followed by brain evolution [20], [46]–[48]. The primary evidence for this hypothesis has been that the slope of a regression of brain volume on body size is much greater among distantly- than closely-related species [1], [20], [49]. If the relationship between brain and body size arose solely due to selection on body size, the two slopes should be the same [47], but they are not. Brain size has been observed to change more slowly than body size among closely related species pairs in which one species exhibits rapid recent body size change, such as those containing ‘phyletic dwarfs’ (e.g. [50]). However, there is currently no empirical evidence directly supporting the hypothesis that such lags persist over long evolutionary time periods or that this operates as a general mechanism in brain:body size evolution [51]. In fact, the only test of the ‘lag’ hypothesis across a large taxonomic group, the primates, failed to support this hypothesis [8].

Here we use computed tomography (CT) techniques to create virtual brain endocasts from the skulls of 36 terrestrial species in the order Carnivora to assess the relative importance of social, ecological, and life history traits on both overall encephalization and the relative volumes of specific brain regions. Carnivores offer an excellent model for these tests because they exhibit great variation in brain and body size, and their social and physical environments both span broad ranges of complexity. Nevertheless, in part because research on brain evolution still focuses mainly on primates (e.g. [9], [20], [42]) and birds (e.g. [35], [37], [52]) we still lack a complete understanding of the environmental correlates of encephalization in Carnivora (e.g. [17], [18], [53]). Previous work on brain evolution has demonstrated the importance of simultaneously assessing predictors in a framework integrating multiple hypotheses [5], [38]. We therefore implement phylogenetically-corrected generalized least squares (PGLS) models to account for shared evolutionary history, while simultaneously assessing the importance of the social and physical environment in encephalization. Finally, we evaluate the effects of recent evolutionary changes in overall body size on relative brain volume, as well as effects of recent evolutionary changes in overall endocranial volume on the relative volumes of specific brain regions.

## Methods

### Data Collection

#### Details of phylogeny

We used the phylogeny for the order Carnivora presented by Bininda-Emonds et al. [54] due to its broad taxonomic coverage, but supplemented it with updated family-level molecular phylogenies for Felidae [55] and Hyaenidae [56]. Branch lengths are poorly known for Carnivora, so we used Pagel’s arbitrary branch lengths [57], which is probably the most common approach (e.g. [18]). We estimated Blomberg’s k [58], a measure of the degree of phylogenetic autocorrelation among our raw brain measurements using the ‘picante’ package [59] in R v.2.12.1 [60]. Branch length differences generally have little effect on regression analyses [61]. Pagel’s branch lengths were calculated in Mesquite [62] using the PDAP:PDTree module [63]. All other transformations and analyses were performed in R.

#### Specimens and measurements

Skull specimens from multiple adult members of each of 36 carnivore species were obtained from the collections of the Michigan State University Museum, Field Museum of Natural History, Natural History Museum of Los Angeles County, National Museum of Natural History, and University of Michigan Museum of Zoology (see Table S1). We used mean values from each species, averaging male and female values when they differed (Table S2).

All skulls were scanned using a General Electric Lightspeed 4 slice CT or General Electric Discovery ST 16 slice CT scanner in the Department of Radiology at Michigan State University. Each skull was aligned in the scanner rostrocaudally to replicate the natural anatomical position of the head. Parameters for each scan were as follows: 0.625 mm slice thickness, 30 cm field of view, 5.62 mm/rotation table speed, and 0.562∶1 pitch. CT images were saved in DICOM (Digital Imaging and Communications in Medicine) Centricity Version 2.2 format. Virtual endocasts were created using the software package MIMICS 11.02 (Materialise, Inc., Ann Arbor, MI, USA). The skull was separated from air space by setting a grayscale pixel value as the threshold for filling in the endocranial air space. This space was filled in each coronal slice starting rostrally where the cribiform plate forms the floor of the intracranial cavity and continuing caudally through the foramen magnum. The resulting coronal sections were combined to create a three-dimensional reconstruction (virtual endocast) of the intracranial cavity using the MIMICS 3D object operation. Smoothing algorithms were applied to enhance the image and eliminate uneven surfaces. Detailed external brain morphology, including gyral and sulcal patterns, could be seen clearly in all virtual endocasts utilized here [64].

Skull basal length was defined as the distance from the anterior border of the median incisive alveolus to the mid-ventral border of the foramen magnum. Skull height was measured as the greatest height of the cranium perpendicular to the plane of the basioccipital and basisphenoid bones ([65]: measurement 18), excluding the sagittal crest, when one was present [66]. Finally, zygomatic arch breadth was measured as the greatest width of the skull. A single observer (B.M.A) collected all linear skull measurements from the CT images. Species means for skull measures are given in Table S2.We used species-wide averages obtained for body mass from the literature because we did not have individual mass values for many of the specimens in our data set, and wanted to avoid introducing two separate sources of error (see Table S3).

#### Virtual Endocasts (VE)

Assessments of total endocranial and regional volumes were obtained using the MIMICS 3D volume measurement operation. Total endocranial volume was defined as the volume extending from the rostral tip of the olfactory bulbs caudally to the foramen magnum. CT files were coded by animal number only, and analysis and demarcation of brain regions were conducted blind with regard to the identity of individuals or species. All volumetric measurements and analyses were made by a single observer (B.M.A). Although relevant comparisons among carnivorans have not been published, previous work suggests that differences between endocranial and brain volumes are either very small [67] or nonexistent [68]. Moreover, our CT measures of endocranial volume were comparable to actual brain volume measurements from Rohrs’ data [69], [70] given in Dunbar and Bever [71] (paired t-test: *t* = 0.826, number of species pairs  = 12, *p* = 0.425). We also compared the log transformed endocranial volumes based on the present CT method with previously reported log transformed brain volumes primarily estimated using the bead method [17] and found no difference (*t* = 0.320, number of species pairs *n* = 29, *p* = 0.751). Because the species compositions of these two comparisons were different, log transformation was used to improve normality for the second comparison, but not the first. Log transformation did not qualitatively affect the result in either case.

**Figure 1:**
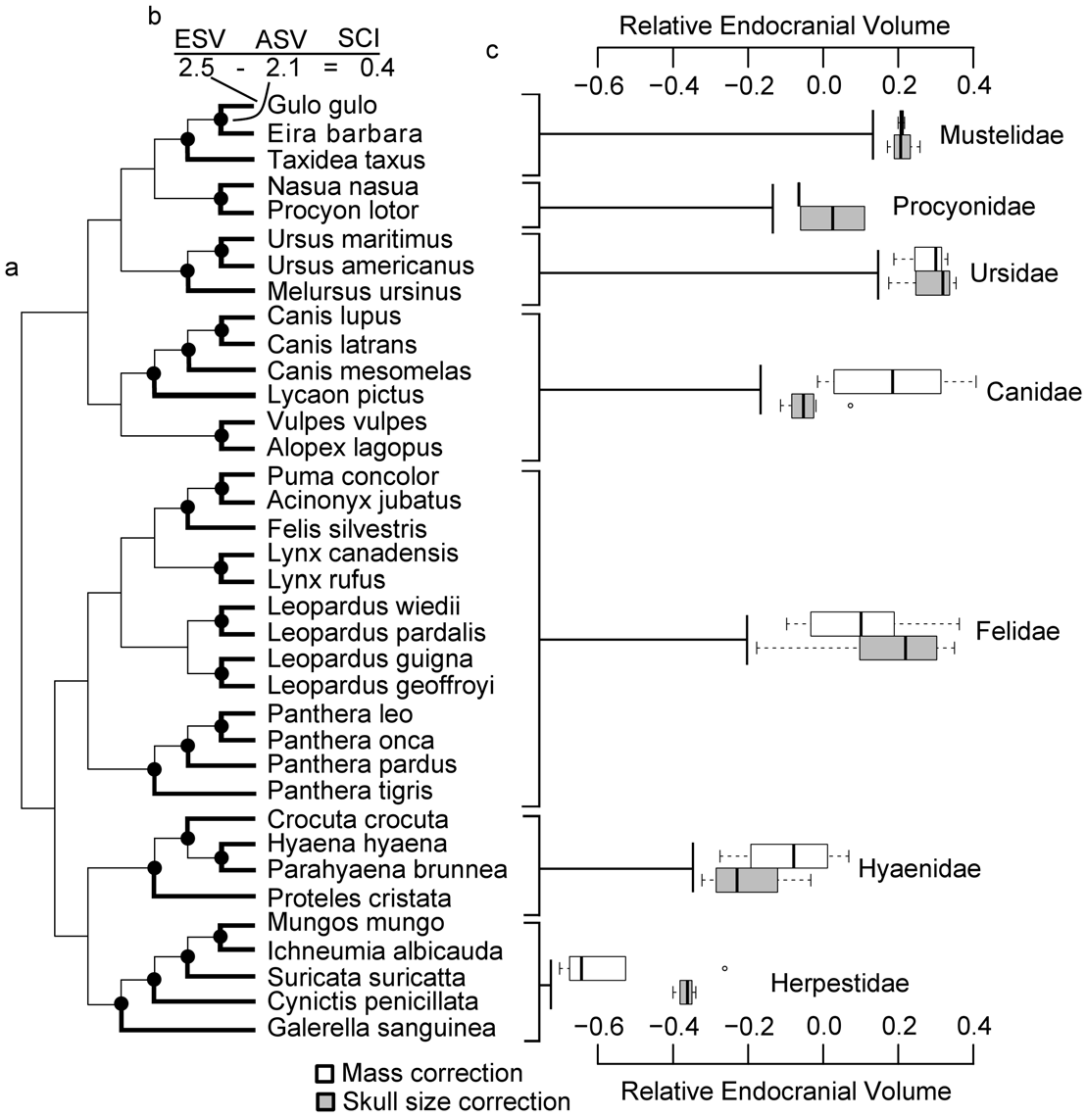
Carnivore phylogeny, demonstration of size-change indices, and relative endocranial volumes by family. a) Carnivore phylogeny with Pagel’s arbitrary branch lengths. Filled circles represent the hypothetical ancestors or nodes at which the ancestral traits were estimated. Heavy lines link each extant species to the ancestral node that was subtracted from the value for the extant species to obtain size change indices (SCIs). b) Demonstration of how SCIs were calculated. Most recent estimated ancestral size values (ASV) were subtracted from the associated value for extant species size (ESV), and the difference is equal to the SCI. c) Box-and-whisker plot displaying degree of variation in relative brain size within each family. Relative MCOEV is indicated by a white box and relative SCOEV by a grey box. Boxes indicate interquartile range, and whiskers spread to the furthest points outside the interquartile range, but within 1.5 times the interquartile range from the median.

For each endocast, sulcal patterns and/or bony landmarks were used to delineate 3 principal regions: anterior cerebrum (Ac) as a measure of frontal cortex, posterior cerebrum (Pc) as a measure of the remaining cerebrum, and the combination of the cerebellum and the brainstem (Cb+Bs) as an approximate rhombencephalic measure. For the volumetric analysis, anterior cerebrum (Ac) was defined as the region rostral to the junction of the cruciate sulcus and midline, and caudal to the olfactory bulbs. For species lacking cortical maps, we relied on identification of the anterior cortical areas in other carnivores, and applied the same criteria. In primates, frontal cortex includes cortex rostral to the central sulcus, but the likely homologue to the central sulcus in carnivorans is too subtle to provide a suitable landmark to use in endocasts [27]. The boundary between motor and somatosensory cortex is the post-cruciate sulcus, which is not reliably present in all carnivores [27]. Instead, the cruciate sulcus was used as a landmark for demarcating brain regions, as this is a prominent feature that demonstrates less intra- and interspecific variation than the post-cruciate sulcus [72]. The cruciate sulcus is coincident with the rostral-most portion of motor cortex in the cat (*Felis catus*: [73]), dog (*Canis lupus*: [74]), and raccoon (*Procyon lotor*: [75]). In each of these species, the dorsal bank of the cruciate sulcus is coincident with cytoarchitectonic area 4. This has not been confirmed for other carnivoran families, but seems likely given the phylogenetic spread of the families for which it has been confirmed (see Fig. 1 for families included in our analysis). Anterior cerebral volume was calculated from the endocranial slices and was thus comprised of frontal cortex and subcortical structures, including a small portion of the rostral-most head of the caudate nucleus, ventral pallidum, olfactory tubercle and prepiriform cortex. Posterior cerebrum (Pc) included the endocranial volume posterior to the cruciate sulcus, but anterior to the tentorium cerebelli. Lastly, the cerebellum and brainstem (Cb+Bs) are housed within the intracranial cavity in the posterior cranial fossa; this region was defined as the portion of the intracranial cavity that begins at the most anterior border of the tentorium cerebelli and extends posteriorly to the foramen magnum of the occipital bone.

#### Transformations and brain variables

Total endocranial volume included all of the measures of interest, as well as the olfactory bulbs, which were not used in other analyses. Overall endocranial volume relative to body size was calculated in two different ways, each using a different correction factor. First, we calculated overall endocranial volume relative to species mean body mass. Second, we calculated overall endocranial volume relative to the size of the particular skull from which each virtual endocast was generated; skull size was calculated as the first principal component axis (PC1) from a phylogenetically-corrected principal component analysis (PCA) on three skull measurements: basal length, skull height, and zygomatic arch breadth [76]. Here we use the acronym OEV to represent overall endocranial volume. Thus mass-corrected OEV is noted as MCOEV and size-corrected OEV as SCOEV. MCOEV and SCOEV were calculated as residuals from phylogenetically-corrected regressions of endocranial volume on body mass and skull size, respectively, by regressing endocranial volume on each measure using PGLS (PGLS: [77]–[80]).

In addition to OEV, we also estimated the volume of each of the measured brain regions relative to OEV, including cerebrum anterior to the cruciate sulcus (Ac), cerebrum posterior to the cruciate sulcus (Pc), total cerebrum (Ac+Pc), and hindbrain, which includes both cerebellum and brainstem (Cb+Bs). The relative volume of each brain region was calculated by taking residuals from a regression of the brain region on ‘brain rest’ (overall endocranial volume minus the volume of the region of interest; e.g. [40], [71]); the resulting variables are henceforth referred to as the “relative” region (e.g. relative Ac, Pc, Cb+Bs, total cerebrum). All morphological data were log-transformed prior to analysis.

#### Ancestral reconstruction and SCI calculation

We performed maximum likelihood (ML) ancestral reconstruction [81] on multivariate skull size, body mass and endocranial volume using the ‘geiger’ package in R [82]. Indices of recent evolutionary size change (SCIs) were calculated by subtracting a reconstructed ancestral species value (ASV) from an extant species value (ESV). For this calculation, we used the value reconstructed for the node representing the most recent hypothetical ancestor shared by any other extant species in the phylogeny (see [83]). This calculation represents the magnitude of change rather than the rate, bypassing the problem associated with using arbitrary branch lengths when calculating rate of change.

#### Social, ecological and life history variables

We collected social, life history and ecological data from a variety of sources (detailed in Table S3). We used these variables to generate one composite variable representing social complexity and another representing life history traits. Ecological variables were not combined because we had no a priori expectation that those variables were conceptually related. For the variable representing social complexity, group size, feeding/hunting group size and degree of social cohesion were included in a phylogenetically-corrected PCA [76], and each species’ score from the first principal component axis (PC1) was used as a composite measure of social complexity. A composite measure of sociality is useful because it has been suggested by a number of sources that complexity of the social system is more important than the number of individuals, especially given the high prevalence of diffuse grouping patterns [37], [84], [85]. A composite measure provides information not only on the size of groups, but their complexity, encompassing the degree of social cohesion and possible interaction during feeding/hunting. Similarly, age at weaning, gestation length, and longevity were included as life-history variables in a phylogenetically corrected PCA to calculate an overall life history axis for the sample (PC1). Life history variables have widely been documented as varying strongly along a multivariate axis, often termed the ‘fast-slow’ life history axis, which explains a great deal of life history variation even after correcting for body size [86]–[92]. Life history variables were corrected here for skull size, used as a proxy for overall body size, before performing the PCA. Ecological variables included diet (primarily carnivorous, insectivorous or omnivorous) and log home range size corrected for skull size. Finally, we scored species on an index describing degree of forepaw use in food processing (see Table S3). All data were collected in accordance with National Institutes of Health guidelines, and have been approved under Michigan State University’s Animal Care and Use Protocol #AUF 07/-08-099-00.

### Data Analysis

#### Influence of phylogeny

We estimated Blomberg’s k for each relative brain measure using Pagel’s branch lengths. In addition, Moran’s I, a general measure of autocorrelation, was estimated at three different taxonomic levels (suborder, family, genus) for each relative brain measure [93]–[95]. Moran’s I was calculated on cophenetic distances among traits, both for the entire phylogeny and at lower taxonomic levels, using the R package ‘ape’ [96].

#### PGLS analysis

Phylogenetic comparative methods are potentially powerful tools for studying adaptation that are now commonly used to avoid the problem of phylogenetic non-independence [97]–[99]. Although there are alternative methods for addressing this problem, PGLS techniques allow simultaneous consideration and estimation of the degree of phylogenetic non-independence using Pagel’s lambda (λ). λ describes a continuous variable in which zero represents a trait that displays no phylogenetic signal and one describes a trait that has evolved under brownian motion. We used PGLS to fit a series of six models with the ecological, social and life history variables of interest as predictor variables, as well as diet (see Table S3 for variables included). We fit each of the six models three different ways: fixing λ to zero, allowing it to assume its maximum likelihood estimate, and fixing it to one. Model selection was performed using sample-size corrected Akaike’s Information Criterion (AICc). To avoid overfitting, a potential consequence of stepwise model selection procedures [100], we estimated only full models, comparing models that vary only in the degree of phylogenetic autocorrelation among residuals. Thus every model we estimated contained all possible predictor variables.

## Results

### Effect of Phylogeny

**Figure 2:**
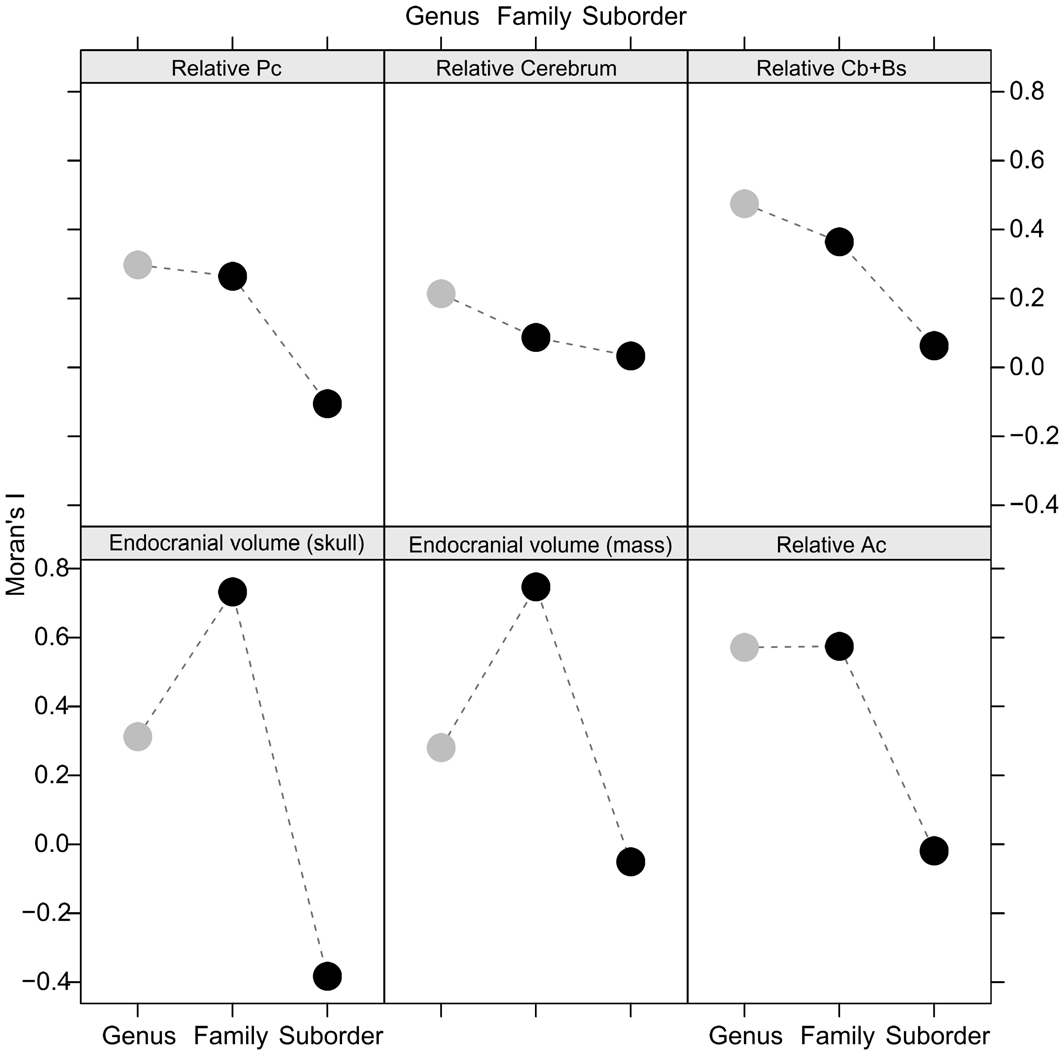
Phylogenetic autocorrelation as measured using Moran’s I, which ranges between −**1 and 1.** Black circles indicate statistically significant autocorrelation at α≤0.05, and grey circles indicate measures that are not significant at α≤0.05.

Phylogenetic autocorrelation based on Blomberg’s k was strong and statistically significant for both measures of relative overall endocranial volume, and moderate and significant for relative Ac volume and relative Cb+Bs volume (Table 1). The strength of phylogenetic signal was moderate for relative Pc and relative total cerebrum volume, but did not differ significantly from zero. Estimates of phylogenetic signal using Moran’s I generally supported the results obtained using Blomberg’s k (Fig. 2). Specifically, traits that exhibited strong, statistically significant phylogenetic signal using Blomberg’s k also did so using Moran’s I, but this pattern was clear only at the taxonomic level of family, suggesting that phylogenetic autocorrelation at or below the level of family drives the observed overall phylogenetic autocorrelation. Autocorrelation at the level of genus was non-significant, even when of large magnitude, although this may simply reflect the small sample sizes within most genera in our data set (Fig. 1). Finally, autocorrelation at the level of suborder was statistically significant for all brain measures, with Moran’s I close to 0 for all except SCOEV, for which it was negative (Fig. 2). Negative autocorrelation is exhibited by traits for which closely related species differ more than do distantly related species, and can result from character displacement [101].

**Table 1:** Phylogenetic autocorrelation among brain measures.

	*K*	*z*	*p*
**MCOEV**	1.188	−4.036	<0.001
**SCOEV**	1.169	−4.212	<0.001
**Relative Ac**	0.774	−3.293	<0.001
**Relative Pc**	0.4	−0.862	0.207
**Relative Cb+Bs**	0.491	−1.821	0.027
**Relative Cerebrum**	0.364	−0.415	0.367

Degree of phylogenetic autocorrelation in relative brain volume measures using Blomberg’s k. K is the degree of phylogenetic signal, Z is the position in the Z distribution estimated from a tip rearrangement test using 100,000 iterations, and p is the p-value estimated from the tip rearrangement test.

### Composite Social Complexity and Life History Variables

The first principal component (PC) axis resulting from the PCA on the three social variables used as our proxy for social complexity, explained 78.5% of the variance. All three univariate social variables exhibit strong positive loadings with PC1 (social group size: 0.932, feeding group size: 0.764, social cohesion: 0.950), indicating a large contribution from each. The composite life history variable (the scores from the first PC axis) resulting from the PCA on gestation length, weaning age, and maximum lifespan explained 44.2% of the variance in those data. Gestation length, weaning age, and maximum lifespan all increase with PC1 (loadings were 0.828, 0.499, and 0.627 respectively). As has been documented in many studies on life history covariation (e.g. [86], [91], [92], [102], [103]), the first multivariate axis of covariation among life history variables in our data appears to correspond to a ‘fast-slow’ life history axis, where species develop more slowly, reproduce more slowly, but live longer as PC1 increases. This ‘fast-slow’ axis is observed both with raw life history variables and also after correcting for mass [86], [89], [91], [92], [102], [103].

### Overall Encephalization

**Figure 3:**
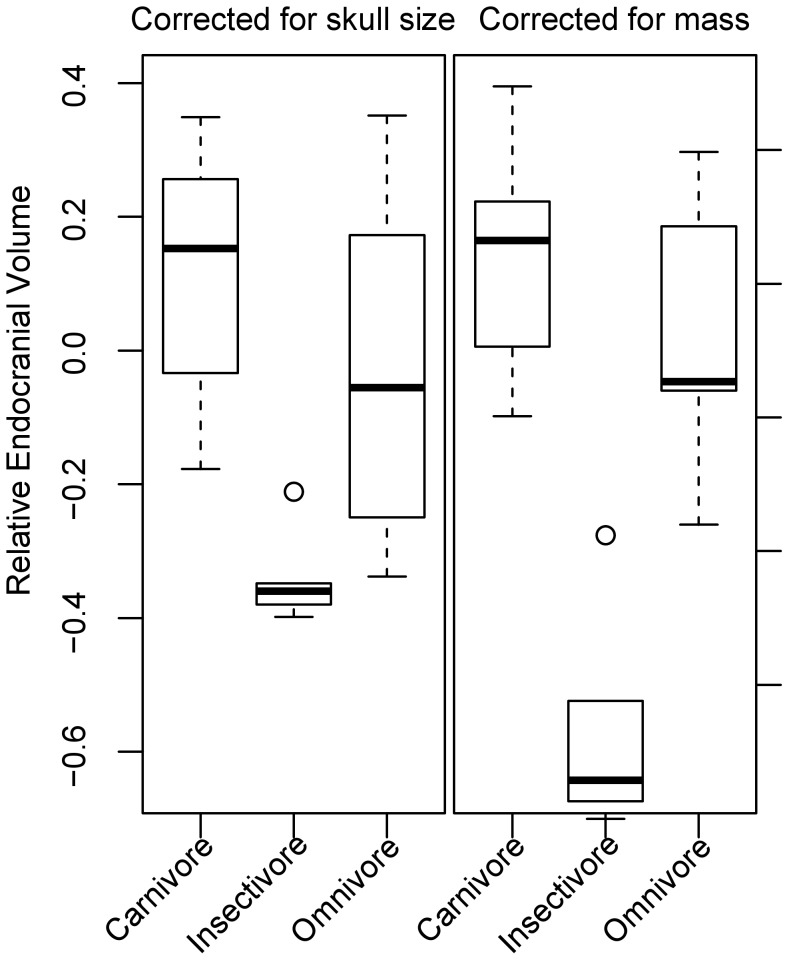
Box plot showing relationship between diet and relative endocranial volume. Boxes indicate interquartile ranges, and whiskers spread to the furthest points outside the interquartile range, but within 1.5 times the interquartile range from the median.

For the PGLS regressions and ANOVA, we present in each case the results from the best model, chosen from models with lambda fixed at zero or one, or allowing lambda to take its MLE (see Table S4). After accounting for phylogeny, both measures of relative endocranial volume were influenced by diet (Fig. 3; Corrected for skull size: *F*_2,28_ = 4.57, *p* = 0.019, Corrected for mass: *F*_2,28_ = 12.01, *p*<0.001). Flesh-eating species had the largest relative endocranial volumes, omnivores were intermediate, and insectivores had the smallest relative brain volumes, although the difference between insectivores and omnivores was statistically significant only for MCOEV (Table 2). Both MCOEV and SCOEV were negatively related to recent changes in the respective body size measure (SCIs), suggesting an evolutionary lag, during which body size has evolved, but brain size has yet to catch up and return to the basal brain:body allometry (Table 3).

**Table 2:** Effects of diet on relative endocranial measures.

Brain region	Comparison	*ß*	*SE*	*T*	*p*
**MCOEV**	**I vs. C**	−0.586	0.13	−4.506	**<0.001**
	**O vs. C**	−0.251	0.076	−3.306	**0.003**
	**I vs. O**	−0.335	0.127	−2.638	**0.014**
**SCOEV**	**I vs. C**	−0.308	0.129	−2.392	**0.024**
	**O vs. C**	−0.193	0.075	−2.567	**0.016**
	**I vs. O**	−0.115	0.124	−0.928	0.361
**Cerebrum**	**I vs. C**	−0.134	0.073	−1.822	0.079
	**O vs. C**	−0.051	0.043	−1.183	0.247
	**I vs. O**	−0.083	0.068	−1.222	0.232
**Ac**	**I vs. C**	0.216	0.444	0.487	0.63
	**O vs. C**	−0.043	0.264	−0.164	0.871
	**I vs. O**	0.26	0.421	0.616	0.543
**Pc**	**I vs. C**	−0.286	0.23	−1.243	0.224
	**O vs. C**	−0.018	0.17	−0.108	0.915
	**I vs. O**	−0.267	0.219	−1.224	0.231
**Cb + Bs**	**I vs. C**	0.15	0.085	1.762	0.089
	**O vs. C**	−0.046	0.069	−0.675	0.505
	**I vs. O**	0.196	0.088	2.232	**0.034**

Effects of diet from ANOVAs, and contrasts from multiple regressions. For the contrasts, C represents carnivore, O represents omnivore and I represents insectivore. In each case, the first species is the one being contrasted. For example, I vs. C means that the estimate of effect under β is the change in the response variable due to insectivory with carnivores as the intercept. Note that diet effects were estimated as part of the multiple regression results in Table 3, and are in a separate table only for presentation purposes.

**Table 3:** PGLS regression outputs for all variables other than diet.

Trait	Predictor	*ß*	*SE*	*T*	*p*
**MCOEV**	**Intercept**	0.110	0.129	0.853	0.401
	**Body SCI**	−0.102	0.034	−3.045	**0.005**
	**Sociality**	−0.025	0.027	−0.919	0.366
	**Home Range**	−0.005	0.015	−0.350	0.729
	**Forelimb Use**	0.022	0.053	0.405	0.689
	**Life History**	0.039	0.037	1.045	0.305
**SCOEV**	**Intercept**	0.003	0.126	0.026	0.979
	**Brain SCI**	−0.170	0.074	−2.295	**0.029**
	**Sociality**	−0.046	0.026	−1.756	0.090
	**Home Range**	0.000	0.015	−0.023	0.982
	**Forelimb Use**	0.050	0.052	0.958	0.346
	**Life History**	0.029	0.036	0.790	0.436
**Cerebrum**	**Intercept**	0.067	0.045	1.470	0.153
	**Brain SCI**	−0.032	0.063	−0.501	0.620
	**Sociality**	0.081	0.022	3.711	**0.001**
	**Home Range**	0.017	0.015	1.143	0.263
	**Forelimb Use**	−0.014	0.024	−0.555	0.583
	**Life History**	−0.004	0.020	−0.224	0.825
**Ac**	**Intercept**	0.989	0.427	2.314	0.028
	**Brain SCI**	0.573	0.233	2.464	**0.020**
	**Sociality**	−0.036	0.088	−0.404	0.689
	**Home Range**	−0.117	0.051	−2.305	**0.029**
	**Forelimb Use**	−0.554	0.178	−3.120	**0.004**
	**Life History**	0.034	0.122	0.278	0.783
**Pc**	**Intercept**	−0.285	0.209	−1.363	0.184
	**Brain SCI**	−0.163	0.156	−1.047	0.304
	**Sociality**	0.061	0.063	0.961	0.345
	**Home Range**	0.067	0.035	1.891	0.069
	**Forelimb Use**	0.183	0.102	1.785	0.085
	**Life History**	0.043	0.080	0.540	0.594
**Cb + Bs**	**Intercept**	−0.092	0.076	−1.214	0.235
	**Brain SCI**	0.084	0.080	1.050	0.303
	**Sociality**	−0.106	0.031	−3.487	**0.002**
	**Home Range**	−0.031	0.018	−1.690	0.102
	**Forelimb Use**	0.048	0.041	1.171	0.252
	**Life History**	0.043	0.030	1.419	0.167

Multiple regression output for the best model for each of the different response variables, not including effects of diet, because diet is categorical. Body SCI is the size change index for body mass or skull size. Brain SCI is the size change index for brain volume. Sociality is PC1 from the PCA of the variables describing social complexity. Home range is log home range size corrected for body size in the same way as brain volume. Forelimb use is our measure of forelimb dexterity. Finally, life history is PC1 from a PCA of the three life history variables we included in our analysis.

### Regional Brain Volumes

Sociality was positively related to relative total cerebrum volume, and negatively related to Cb+Bs volume, but not significantly related to other brain measures (Table 3). Both forelimb use and home range size predict relative Ac volume negatively, and exhibit nearly significant positive trends with Pc (Table 3). The composite life history variable was not significantly related to any response variable. Although the effect of diet on relative Cb+Bs volume was observable only as a non-significant trend (*F*_2,28_ = 2.549, *p* = 0.097), insectivores have significantly larger relative Cb+Bs than omnivores (Table 2). Diet did not have an influence on the relative volume of the other brain regions (Cerebrum: *F*_2,28_ = 1.77, *p* = 0.189; Ac: *F*_2,28_ = 0.19, *p* = 0.828; Pc: *F*_2,28_ = 0.889, *p* = 0.422; for contrasts see Table 2) Finally, relative Ac volume was positively associated with recent evolutionary changes in brain size (indicated by SCI values), suggesting that increased overall encephalization has been accompanied by a disproportionate increase in size of the frontal brain in mammalian carnivores (Table 3).

## Discussion

Although sociality plays an important role in primate brain evolution [42], our data failed to support the social brain hypothesis as an explanation for overall encephalization in Carnivora. Previous studies have demonstrated a relationship between relative brain volume and sociality in this order (e.g. [18], [71]), but those findings are controversial. In particular, Perez-Barberia et al. [18] suggested that carnivore species with large brains for their body size are more commonly social than other species. However, Finarelli and Flynn (2009) showed that this trend in the Perez-Barberia et al. data disappeared when the family Canidae was removed from the analysis, and noted further that both Ursidae and Mustelidae are largely asocial, yet relatively large-brained. Although our results did not support the social brain hypothesis as it pertains to overall brain volume, we did identify a positive relationship between relative cerebrum volume and sociality (Table 3). This relationship suggests that simply excluding the brain stem, cerebellum and olfactory bulbs, regions of the brain that are likely to be less critical for social cognition, allows us to identify a pattern not observed using relative endocranial volume. Interestingly, the opposing relationship demonstrated between relative cerebellum volume and sociality most likely suggests that either reduced cerebellum and brain stem volume accompanies increased sociality, or there are additional factors opposing increases in overall brain volume, thus necessitating a decrease in cerebellum and brain stem volume with increases in cerebrum volume. The factors most likely to act to constrain evolutionary increases in brain size are selection to maintain skull size or shape, or antagonistic selection on overall brain size due to the energetic costs of neural tissue [30].

Although we failed to demonstrate a relationship between sociality and overall encephalization in Carnivora, we did identify a relationship between overall encephalization and diet (Table 2). Gittleman [53] had suggested this relationship, but was unable to support it statistically. In our data set, species that are primarily carnivorous have larger relative endocranial volumes than omnivorous or insectivorous species, and omnivorous species have larger relative endocranial volumes than insectivorous species, though the latter difference is statistically significant only when endocranial volume is corrected for mass, not skull size (Table 2). Two main hypotheses have been put forward to explain the relationship between diet and degree of encephalization. The first is that some diets are more energetically efficient than others, allowing the evolution of metabolically expensive brain tissue [30], [31]. The second is that some diets require more complex cognitive processing to acquire and process food items [9], [32].

In addition to its relationship with overall encephalization, diet also appears to be related to the relative volume of one of our measured brain regions, the cerebellum and brain stem, which is significantly larger in insectivores than in carnivores (Table 2). Other ecological traits are also associated with the volume of specific brain regions. The relative volume of the cerebrum anterior to the cruciate sulcus (Ac) is negatively related to home range size and degree of forelimb use in food processing, but positively related to the magnitude of recent size change in overall brain volume (Table 3). Our Ac measure consists primarily of frontal cortex. In primates, frontal cortex has been implicated in social cognition and executive function [104]. Interestingly, recent work has revealed a relationship between Ac and social group size within the family Hyaenidae [64], a finding not supported by our larger data set representing the entire order Carnivora. This suggests that some of the patterns observed here might be evident among species in broader taxonomic groups, whereas other patterns may become apparent only when considering species at lower taxonomic levels, such as families.

Estimates of recent evolutionary changes in body size in our data set, indicated by SCI values, were negatively associated with relative brain volume (Table 3). In other words, species in lineages characterized by a recent increase in body size have relatively small brains for their body size, while those in lineages characterized by a recent decrease in body size have relatively large brains for their body size. Thus, our results provide the first direct empirical support for the ‘lag’ hypothesis over longer evolutionary time periods, which suggests that brain and body size co-evolve, but that body size changes first, followed later by changes in brain size that return the relationship to its basal allometry [1], [8], [20], [47]. Interestingly, an earlier analysis using slightly different methods found no relationship between SCIs and brain volume in primates [8]. Although it may be that the lag is simply weaker or absent among primates, it is also possible that the pattern becomes evident only when other important variables, such as diet, are accounted for statistically. The eventual changes in brain size required to return to the basal brain:body size allometry observed among distantly related species may occur either due to selection directly on brain size, or to changes in the genetic and developmental mechanisms underlying body size evolution at greater taxonomic distances [105].

A similar relationship, though positive, was found between relative Ac volume and recent changes in overall brain volume, indicating that increases in brain size result in disproportionate increases in frontal brain. This pattern suggests that developmental mechanisms similar to those suggested by Finlay and Darlington [43] might explain some variation in relative Ac volume. However, the fact that we were able to reveal that specific brain regions are significantly related to ecological and social traits requires that brain regions also evolve independently to some extent. In addition, some environmental factors in our data set are positively related to one brain region but negatively related to another (Table 3). For example, sociality positively predicts relative total cerebrum volume, but negatively predicts Cb+Bs, which represents the remaining endocranial volume minus the olfactory bulbs. Similarly, Ac volume is negatively related to forelimb dexterity and home range size, while Pc volume is positively related to forelimb dexterity and home range size. Although these latter relationships were not statistically significant, the trade-off effect between the Ac and Pc may be due to an increased demand for processing forelimb tactile information in the somatosensory cortex [27] and enhanced spatial memory processing in the hippocampus [106] respectively, within Pc. Since the endocast method does not permit analysis of subcortical features, we were unable to separate the relative contributions of these areas within Pc. Such tradeoffs among regions of the brain have been seen in previous studies [42], and suggest that increases in some brain regions are accompanied by concomitant decreases in other brain regions. These patterns are suggestive of negative microevolutionary tradeoffs among brain regions due to either space or energetic limitations, and indicate that variation in overall encephalization might reflect not only selection to increase the volume of the brain, but also antagonistic selection acting to oppose further encephalization.

Interestingly, the high degree of variation apparent among families within the order Carnivora suggests that there are some factors operating at the level of the family that prevent or slow evolutionary change, at least over the time scale considered here. Specifically, species within some families share large brains or bodies, whereas species in others share small brains or bodies (Fig. 1), and significant phylogenetic autocorrelation appears to be highest at the family level for most traits (Fig. 2). In many cases, intrafamily variation in morphological and ecological traits is fairly low, resulting in clear diagnostic characters for some families. Additionally, it is clear from our data set that much of the variation in several different measures is explained simply by the family to which a species belongs. This strong family-level variation echoes our earlier suggestion that analyses within families may in some cases uncover clearer patterns than analyses targeting broader taxonomic ranges.

Selection of a body size correction factor for use in studies of brain size is clearly a more complicated issue than is commonly assumed. Although correcting for skull size or mass yielded similar results in our regression analyses, some differences were apparent. For instance, canids have much larger values for MCOEV than SCOEV, whereas the converse is true for herpestids. Although there are arguments in favor of using both mass and skull size as correction factors, the proper correction factor depends on why brain volume covaries with body size, which is not known. Until we understand why brain volume scales to body size, the best scaling factor cannot be known unequivocally, if there is in fact a ‘best’ scaling factor. It is therefore worth noting that most studies do not replicate their analyses using more than one measure of body size. Although such replication may complicate analyses of the environmental predictors of encephalization, more explicit consideration of the size measure used as a correction factor is clearly warranted. Indeed, whether the volume of a brain region is corrected for whole-brain volume or the volume of another specific region of the brain can lead to different conclusions, with very little reason to presume that one correction factor is superior to another [107].

In conclusion, despite some lack of consensus in the study of the evolutionary forces acting on brain volume within the order Carnivora, a number of points are clear from our analysis. Most importantly, several different variables influence encephalization or regional brain volume in our data set, including sociality, ecology, phylogeny and recent evolutionary changes in body size and overall brain size, measured by SCIs. However, the composite life history trait we included in our analysis was unrelated to encephalization or the volume of any brain region (Table 3). Previous analyses have found relationships within Carnivora between encephalization and neonate mass, but not weaning age or gestation length [7], and our results support these conclusions. It is clearly important to consider multiple factors simultaneously, including not only those that are most likely adaptive, but also those that may have no adaptive value. In addition, it is interesting that analyses even of large, fairly crude subdivisions of overall brain volume (e.g. ‘total cerebrum’) can reveal relationships not found in analyses of whole brains. This may be explained by a combination of mosaic processes, developmental linkages and external factors such as antagonistic selection on skull size and shape constraining brain evolution. Thus, although total brain size may not solely reflect selection pressures on specific brain regions, the effects of such selection are still seen in large subdivisions of the brain. Our results provide at least circumstantial evidence that processes leading to concerted change throughout the brain, and those influencing only specific brain regions, both play a role in brain evolution among mammalian carnivores.

## Supporting Information

Table S1 **Details for specimens used in analysis.** Field Museum (FMNH); Los Angeles County Museum of Natural History (LACM); Michigan State University Museum (MSUM); University of Michigan Museum of Zoology (UMMZ). All skulls were scanned using a General Electric Lightspeed 4 slice CT or General Electric Discovery ST 16 slice scanner in the Department of Radiology at Michigan State University. Scanner type is indicated in the final column.
(PDF)

Table S2 Cranial and endocranial measures used in analysis including: endocranial volume in mm^3^ (brain volume), combined cerebellum and brainstem volume in mm^3^ (Cb + Bs), cerebrum anterior to the cruciate sulcus in mm^3^ (Ac), cerebrum posterior to the cruciate sulcus in mm^3^ (Pc), skull basal length in mm (BL), zygomatic arch breadth in mm (ZB), skull height in mm (SH). Details of measurements are given in the methods section of the main document. BL, ZB and SH were included in a principal components analysis to create the skull size variable used for analyses. Cerebrum volume is equal to Ac + Pc.
(PDF)

Table S3 Data used in analysis including: group size (GS), social cohesion (Cohesion), FGS (Feeding group size), mass (in kg), gestation length (Gest. Len.; in days), weaning age (WA; in months), maximum recorded longevity (Longevity; in years), home range (in sq. km), diet, and degree of forelimb processing of food (Forelimb). Group size and home range size are arithmetic species means excluding any values that are more than 5 standard deviations from the mean value to avoid including extremely influential outliers. Social cohesion is scored as either a 1 (solitary except during the breeding season), 2 (primarily pair-living), 3 (fission-fusion sociality) or 4 (obligately social). Mass was taken as the mean value given, pooling males and females. Gestation length is given as the mean value, excluding periods of delayed implantation (embryonic diapause). Diet was coded as primarily insectivorous, carnivorous or omnivorous. Finally, degree of forelimb use during food processing was coded based on descriptions of hunting or food processing, or where unavailable, on the type of food consumed. Forelimb processing was scored from 1 to 4, with 1 being no use of forelimbs in food processing, 2 representing use of forepaws with no grasping or independent use of digits, 3 representing grasping behavior and fairly complex using during processing, and 4 representing highly dextrous use of forepaws during food processing including grasping behavior (only raccoons were placed in this category among our sample of species as per [36]). Superscripts indicate the source of the data: ^1^Wilson and Mittermeier [108]; ^2^Sunquist and Sunquist [109]; ^3^Watts [110]; ^4^Nowak et al. [111]; ^5^Holekamp and Dloniak [112]; ^6^Mech [113]; ^7^Baker [114]; ^8^Calculated from family-specific regression on mass. ^9^Gestation length for sea otters was taken from the AnAge database [115], ^10^Mills and Hofer [116]. A subscript next to a column header indicates that all values in the column specified are taken from the source noted unless otherwise specified in an individual cell. A subscript in the ‘Genus and species’ column indicates that all values in the row specified are taken from the source noted unless otherwise specified in an individual cell or next to a column header.
(PDF)

Table S4 **Model selection for best PGLS models, comparing models fitting a fixed brownian motion model (equivalent to using independent contrasts), a fixed ‘no effect of phylogeny’ model, and a model allowing lambda to take its MLE.**
(PDF)
