# Supplementary material for: Multiple Determinants of Whole and Regional Brain Volume among Terrestrial Carnivorans
Source: PLoS One. 2012 Jun 13;7(6):e38447. doi: 10.1371/journal.pone.0038447 (PMC3374790; doi:10.1371/journal.pone.0038447)
Supplement: Table S1 — Details for specimens used in analysis. Field Museum (FMNH); Los Angeles County Museum of Natural History (LACM); Michigan State University Museum (MSUM); University of Michigan Museum of Zoology (UMMZ). All skulls were scanned using a General Electric Lightspeed 4 slice CT or General Electric Discovery ST 16 slice scanner in the Department of Radiology at Michigan State University. Scanner type is indicated in the final column. (PDF) [file pone.0038447.s001.pdf]

Table S1

**Location**	**Catalog No.**	**Family**	**Genus and species**	**Sex**	**Scanner**
MSUM	3948	Herpestidae	*Galerella sanguinea*	Unknown	4
MSUM	24285	Herpestidae	*Cynictis penicillata*	Unknown	4
MSUM	24281	Herpestidae	*Suricata suricatta*	Unknown	4
MSUM	9210	Herpestidae	*Ichneumia albicauda*	Unknown	4
MSUM	11574	Herpestidae	*Mungos mungo*	Unknown	4
MSUM	24290	Hyaenidae	*Proteles cristata*	Female	4
FMNH	95920	Hyaenidae	*Proteles cristata*	Female	4
MSUM	24289	Hyaenidae	*Proteles cristata*	Male	4
MSUM	36216	Hyaenidae	*Proteles cristata*	Male	4
FMNH	95919	Hyaenidae	*Proteles cristata*	Unknown	4
MSUM	33836	Hyaenidae	*Parahyaena brunnea*	Male	4
UMMZ	95748	Hyaenidae	*Parahyaena brunnea*	Female	4
FMNH	34586	Hyaenidae	*Parahyaena brunnea*	Unknown	4
LACM	54102	Hyaenidae	*Parahyaena brunnea*	Unknown	4
LACM	60620	Hyaenidae	*Parahyaena brunnea*	Unknown	4
NMNH	296134	Hyaenidae	*Parahyaena brunnea*	Unknown	4
NMNH	429178	Hyaenidae	*Parahyaena brunnea*	Unknown	4
FMNH	34585	Hyaenidae	*Parahyaena brunnea*	Unknown	4
FMNH	140216	Hyaenidae	*Hyaena hyaena*	Female	4
FMNH	140218	Hyaenidae	*Hyaena hyaena*	Female	4
UMMZ	168360	Hyaenidae	*Hyaena hyaena*	Female	4
MSUM	36612	Hyaenidae	*Hyaena hyaena*	Male	4
FMNH	103991	Hyaenidae	*Hyaena hyaena*	Male	4
FMNH	103992	Hyaenidae	*Hyaena hyaena*	Male	4
FMNH	107342	Hyaenidae	*Hyaena hyaena*	Unknown	4
FMNH	140220	Hyaenidae	*Hyaena hyaena*	Unknown	4
FMNH	140219	Hyaenidae	*Hyaena hyaena*	Unknown	4
FMNH	140215	Hyaenidae	*Hyaena hyaena*	Unknown	4
MSUM	13003	Hyaenidae	*Hyaena hyaena*	Unknown	4
MSUM	36556	Hyaenidae	*Crocuta crocuta*	Female	4
MSUM	37623	Hyaenidae	*Crocuta crocuta*	Female	4
MSUM	36581	Hyaenidae	*Crocuta crocuta*	Female	4
MSUM	36567	Hyaenidae	*Crocuta crocuta*	Female	4
MSUM	36550	Hyaenidae	*Crocuta crocuta*	Female	4
MSUM	36568	Hyaenidae	*Crocuta crocuta*	Female	4
MSUM	36553	Hyaenidae	*Crocuta crocuta*	Female	4
MSUM	36570	Hyaenidae	*Crocuta crocuta*	Female	4
MSUM	36569	Hyaenidae	*Crocuta crocuta*	Female	4
MSUM	36165	Hyaenidae	*Crocuta crocuta*	Female	4
MSUM	35856	Hyaenidae	*Crocuta crocuta*	Female	4
MSUM	37465	Hyaenidae	*Crocuta crocuta*	Female	4
MSUM	36083	Hyaenidae	*Crocuta crocuta*	Female	4
MSUM	37628	Hyaenidae	*Crocuta crocuta*	Female	4
MSUM	36551	Hyaenidae	*Crocuta crocuta*	Female	4
MSUM	36008	Hyaenidae	*Crocuta crocuta*	Female	4
MSUM	36011	Hyaenidae	*Crocuta crocuta*	Female	4

(Table S1 continued)

**Location**	**Catalog No.**	**Family**	**Genus and species**	**Sex**	**Scanner**
MSUM	36571	Hyaenidae	*Crocuta crocuta*	Female	4
MSUM	36077	Hyaenidae	*Crocuta crocuta*	Female	4
MSUM	37627	Hyaenidae	*Crocuta crocuta*	Male	4
MSUM	35855	Hyaenidae	*Crocuta crocuta*	Male	4
MSUM	36168	Hyaenidae	*Crocuta crocuta*	Male	4
MSUM	36079	Hyaenidae	*Crocuta crocuta*	Male	4
MSUM	36163	Hyaenidae	*Crocuta crocuta*	Male	4
MSUM	35854	Hyaenidae	*Crocuta crocuta*	Male	4
MSUM	35853	Hyaenidae	*Crocuta crocuta*	Male	4
MSUM	37464	Hyaenidae	*Crocuta crocuta*	Male	4
MSUM	37466	Hyaenidae	*Crocuta crocuta*	Male	4
MSUM	36084	Hyaenidae	*Crocuta crocuta*	Male	4
MSUM	35852	Hyaenidae	*Crocuta crocuta*	Male	4
MSUM	37624	Hyaenidae	*Crocuta crocuta*	Male	4
MSUM	36078	Hyaenidae	*Crocuta crocuta*	Male	4
MSUM	11679	Felidae	*Panthera tigris*	Female	16
MSUM	16626	Felidae	*Panthera tigris*	Male	4
MSUM	11245	Felidae	*Panthera pardus*	Female	4
MSUM	27989	Felidae	*Panthera pardus*	Male	4
MSUM	2876	Felidae	*Panthera onca*	Male	16
MSUM	12243	Felidae	*Panthera onca*	Male	16
MSUM	36073	Felidae	*Panthera leo*	Female	4
MSUM	11241	Felidae	*Panthera leo*	Male	16
MSUM	17866	Felidae	*Leopardus geoffroyi*	Male	4
UMMZ	146504	Felidae	*Leopardus geoffroyi*	Female	16
MSUM	6313	Felidae	*Leopardus guigna*	Female	16
MSUM	2116	Felidae	*Leopardus guigna*	Male	16
MSUM	14529	Felidae	*Leopardus pardalis*	Female	4
MSUM	14593	Felidae	*Leopardus pardalis*	Male	4
MSUM	14609	Felidae	*Leopardus wiedii*	Female	16
MSUM	14727	Felidae	*Leopardus wiedii*	Male	16
MSUM	35210	Felidae	*Lynx rufus*	Female	4
MSUM	35116	Felidae	*Lynx rufus*	Male	4
MSUM	7754	Felidae	*Lynx canadensis*	Female	4
MSUM	25115	Felidae	*Lynx canadensis*	Male	16
MSUM	24298	Felidae	*Felis silvestris*	Female	16
MSUM	24300	Felidae	*Felis silvestris*	Male	16
MSUM	8047	Felidae	*Acinonyx jubatus*	Male	4
MSUM	10658	Felidae	*Puma concolor*	Female	4
MSUM	10659	Felidae	*Puma concolor*	Male	4
MSUM	35978	Canidae	*Alopex lagopus*	Male	4
MSUM	2604	Canidae	*Vulpes vulpes*	Male	4
MSUM	8096	Canidae	*Lycaon pictus*	Unknown	4
MSUM	24250	Canidae	*Canis mesomelas*	Male	4
MSUM	36608	Canidae	*Canis latrans*	Unknown	4
MSUM	36243	Canidae	*Canis lupus*	Female	16
MSUM	37153	Canidae	*Canis lupus*	Female	16
MSUM	35884	Canidae	*Canis lupus*	Male	16

(Table S1 continued)

**Location**	**Catalog No.**	**Family**	**Genus and species**	**Sex**	**Scanner**
MSUM	36245	Canidae	*Canis lupus*	Male	16
MSUM	7948	Ursidae	*Melursus ursinus*	Female	4
MSUM	2766	Ursidae	*Ursus americanus*	Female	16
MSUM	24397	Ursidae	*Ursus americanus*	Male	16
MSUM	9323	Ursidae	*Ursus maritimus*	Male	4
UMMZ	111168	Procyonidae	*Procyon lotor*	Female	16
UMMZ	170525	Procyonidae	*Procyon lotor*	Male	16
MSUM	9350	Procyonidae	*Nasua nasua*	Female	16
MSUM	9346	Procyonidae	*Nasua nasua*	Male	16
MSUM	36607	Mustelidae	*Taxidea taxus*	Male	16
MSUM	9382	Mustelidae	*Eira barbara*	Female	16
MSUM	9377	Mustelidae	*Eira barbara*	Male	16
MSUM	15193	Mustelidae	*Gulo gulo*	Female	16
MSUM	35983	Mustelidae	*Gulo gulo*	Male	16
