# Supplementary material for: Multiple Determinants of Whole and Regional Brain Volume among Terrestrial Carnivorans
Source: PLoS One. 2012 Jun 13;7(6):e38447. doi: 10.1371/journal.pone.0038447 (PMC3374790; doi:10.1371/journal.pone.0038447)
Supplement: Table S2 — Cranial and endocranial measures used in analysis including: endocranial volume in mm3 (brain volume), combined cerebellum and brainstem volume in mm3 (Cb + Bs), cerebrum anterior to the cruciate sulcus in mm3 (Ac), cerebrum posterior to the cruciate sulcus in mm3 (Pc), skull basal length in mm (BL), zygomatic arch breadth in mm (ZB), skull height in mm (SH). Details of measurements are given in the methods section of the main document. BL, ZB and SH were included in a principal components analysis to create the skull size variable used for analyses. Cerebrum volume is equal to Ac + Pc. (PDF) [file pone.0038447.s002.pdf]

Table S2

Family	Genus and species	Common Name	Brain volume	Cb + Bs	Ac	Pc	BL	ZB	SH
Herpestidae	Galerella sanguinea	slender mongoose	8028.91	1551.17	409.60	5766.65	62.75	33.51	24.01
Herpestidae	Cynictis penicillata	yellow mongoose	6949.71	1305.31	579.94	4786.34	60.06	34.19	19.72
Herpestidae	Suricata suricatta	slender-tailed meerkat	7764.49	1155.79	780.23	5550.76	52.50	39.15	23.34
Herpestidae	Ichneumia albicauda	white-tailed mongoose	16644.95	4411.70	4132.24	6895.53	98.74	50.35	30.87
Herpestidae	Mungos mungo	banded mongoose	9654.18	1633.43	724.12	6872.20	70.58	38.79	25.13
Hyaenidae	Proteles cristata	aardwolf	38105.87	8702.96	3742.81	23887.75	127.80	79.80	36.37
Hyaenidae	Parahyaena brunnea	brown hyena	111010.33	21025.30	19624.81	66813.21	216.68	163.83	55.39
Hyaenidae	Hyaena hyaena	striped hyena	104114.12	19004.23	17601.17	63953.03	204.16	150.17	52.75
Hyaenidae	Crocuta crocuta	spotted hyena	160058.94	26474.06	39181.02	90882.65	218.49	165.37	62.17
Felidae	Panthera tigris	tiger	286031.90	54053.33	27764.10	197394.19	255.70	211.02	80.78
Felidae	Panthera pardus	leopard	156612.99	34131.68	12865.86	105649.53	179.24	132.39	56.25
Felidae	Panthera onca	jaguar	156285.91	31758.44	13674.60	106308.74	191.37	160.50	63.37
Felidae	Panthera leo	lion	237224.76	50176.50	31140.69	147047.37	284.90	219.50	77.46
Felidae	Leopardus geoffroyi	geoffroy's cat	36693.61	8214.95	1960.29	25782.06	108.30	76.50	34.99
Felidae	Leopardus guigna	kodkod	28336.98	6280.53	984.01	20437.89	74.97	55.50	33.22
Felidae	Leopardus pardalis	ocelot	67470.57	14264.58	5095.11	46104.63	118.54	87.04	45.13
Felidae	Leopardus wiedii	margay	44833.93	9534.02	2628.23	31674.39	89.79	65.86	39.48
Felidae	Lynx rufus	bobcat	60594.57	12966.96	2668.60	43206.37	107.80	90.50	42.45
Felidae	Lynx canadensis	canadian lynx	78214.42	15330.55	4646.95	56616.17	110.04	93.43	47.48
Felidae	Felis silvestris	european wildcat	38624.51	7800.35	1185.51	28823.62	79.77	65.31	36.61
Felidae	Acinonyx jubatus	cheetah	134907.17	25493.47	5988.94	100028.25	167.42	139.72	60.80
Felidae	Puma concolor	puma	126052.50	25352.62	11808.64	84885.43	160.64	124.37	54.62
Canidae	Alopex lagopus	arctic fox	43341.33	6159.39	7173.54	27496.64	119.60	67.60	38.12
Canidae	Vulpes vulpes	red fox	44812.52	8458.15	7483.57	26307.69	138.15	73.09	39.52
Canidae	Lycaon pictus	African hunting dog	115781.21	18612.38	18443.74	74819.10	182.00	134.30	58.70
Canidae	Canis mesomelas	black-backed jackal	53277.17	8796.65	12404.69	29753.25	146.99	85.40	42.32
Canidae	Canis latrans	coyote	88935.08	13696.98	18405.65	52378.83	194.65	103.80	53.39
Canidae	Canis lupus	grey wolf	132295.37	20617.23	22795.19	83886.02	215.64	134.92	60.75
Ursidae	Melursus ursinus	sloth bear	254812.38	61988.74	38236.39	149274.81	281.42	162.59	72.96
Ursidae	Ursus americanus	black bear	266120.64	53319.28	49704.52	156082.06	251.44	156.92	69.64
Ursidae	Ursus maritimus	polar bear	549997.65	110749.68	82052.20	335323.89	369.80	226.93	98.47
Procyonidae	Procyon lotor	raccoon	41308.47	8043.03	4117.50	27509.96	103.45	72.50	35.39
Procyonidae	Nasua nasua	brown-nosed coati	33923.54	6170.65	7360.51	18553.53	109.60	67.50	34.58

(Table S2 Continued)

Family	Genus and species	Common Name	Brain volume	Cb + Bs	Ac	Pc	BL	ZB	SH
Mustelidae	Taxidea taxus	American badger	57376.21	8470.55	19034.51	27113.09	116.19	76.91	39.20
Mustelidae	Eira barbara	tayra	46022.80	6853.10	5529.80	32578.69	108.57	66.75	38.65
Mustelidae	Gulo gulo	wolverine	74801.15	13090.84	22125.00	36880.82	127.75	100.30	44.81
