# Supplementary material for: Multiple Determinants of Whole and Regional Brain Volume among Terrestrial Carnivorans
Source: PLoS One. 2012 Jun 13;7(6):e38447. doi: 10.1371/journal.pone.0038447 (PMC3374790; doi:10.1371/journal.pone.0038447)
Supplement: Table S3 — Data used in analysis including: group size (GS), social cohesion (Cohesion), FGS (Feeding group size), mass (in kg), gestation length (Gest. Len.; in days), weaning age (WA; in months), maximum recorded longevity (Longevity; in years), home range (in sq. km), diet, and degree of forelimb processing of food (Forelimb). Group size and home range size are arithmetic species means excluding any values that are more than 5 standard deviations from the mean value to avoid including extremely influential outliers. Social cohesion is scored as either a 1 (solitary except during the breeding season), 2 (primarily pair-living), 3 (fission-fusion sociality) or 4 (obligately social). Mass was taken as the mean value given, pooling males and females. Gestation length is given as the mean value, excluding periods of delayed implantation (embryonic diapause). Diet was coded as primarily insectivorous, carnivorous or omnivorous. Finally, degree of forelimb use during food processing was coded based on descriptions of hunting or food processing, or where unavailable, on the type of food consumed. Forelimb processing was scored from 1 to 4, with 1 being no use of forelimbs in food processing, 2 representing use of forepaws with no grasping or independent use of digits, 3 representing grasping behavior and fairly complex using during processing, and 4 representing highly dextrous use of forepaws during food processing including grasping behavior (only raccoons were placed in this category among our sample of species as per [36]). Superscripts indicate the source of the data: 1Wilson and Mittermeier [108]; 2Sunquist and Sunquist [109]; 3Watts [110]; 4Nowak et al. [111]; 5Holekamp and Dloniak [112]; 6Mech [113]; 7Baker [114]; 8Calculated from family-specific regression on mass. 9Gestation length for sea otters was taken from the AnAge database [115], 10Mills and Hofer [116]. A subscript next to a column header indicates that all values in the column specified are taken from the source [file pone.0038447.s003.pdf]

Table S3

Family	Genus and species	Common Name	GS[1]	Cohesion	FGS	Mass[1]	Gest. Len.	WA	Longevity	HR[1]	Diet	Forelimb
Herpestidae	Galerella sanguinea[4]	slender mongoose	1	1	1	0.50	60	1.83	12.6	0.58	O	2
Herpestidae	Cynictis penicillata[4]	yellow mongoose	3.5	3	1	0.81	49	2	15.16	0.65	I	2
Herpestidae	Suricata suricatta[4]	slender-tailed meerkat	6.35	4	6.4	0.75	77	1.75	20.6	5	I	2
Herpestidae	Ichneumia albicauda[4]	white-tailed mongoose	1.15	1	1	4.05	60	2.7	14.8	0.8	I	2
Herpestidae	Mungos mungo[4]	banded mongoose	23.67	4	1	1.38	60.25	1	17.4	0.9	I	2
Hyaenidae	Proteles cristata[4]	aardwolf	1.25	1	1	8.35 [3]	78.33	3.5	18.92	4.5 [1,10]	I	1
Hyaenidae	Parahyaena brunnea[4]	brown hyena	4.8 [1,10]	3	1	42.25 [1,10]	91.75	12	29	308	O	1
Hyaenidae	Hyaena hyaena[4]	striped hyena	2.3 [1,10]	1	1	33.5 [1,10]	90.5	12	23.5	69 [1,10]	O	1
Hyaenidae	Crocuta crocuta[4]	spotted hyena	25.5 [5]	3	6.7 [1,10]	59.1 [1,10]	110	13.5	41	234.5 [5]	C	1
Felidae	Panthera tigris[2]	tiger	1	1	1	200.00	105.62	4.13	26	130.33	C	2
Felidae	Panthera pardus[2]	leopard	1	1	1	46	95.4	3.5	27.3	129.17	C	2
Felidae	Panthera onca[1]	jaguar	1	1	1	72.25	99	4.2	28	75	C	2
Felidae	Panthera leo[2]	lion	8.7	3	5	158	108.77	8.42	27	125.5	C	2
Felidae	Leopardus geoffroyi[2]	geoffroy's cat	1	1	1	4.80	72	2.25	23	6.58	C	2
Felidae	Leopardus guigna[1]	kodkod	1	1	1	2.03	75	1.835[8]	14.3	2.01	C	2
Felidae	Leopardus pardalis[1]	ocelot	1	1	1	10.1	77	3.53	28.2	9.35	C	2
Felidae	Leopardus wiedii[1]	margay	1	1	1	3.65	81	1.87	24	13.45	C	2
Felidae	Lynx rufus[2]	bobcat	1	1	1	9.33	54	2.17	32	59.13	C	2
Felidae	Lynx canadensis[2]	canadian lynx	1	1	1	9.65	62.75	3	26.8	32.5	C	2
Felidae	Felis silvestris[1]	european wildcat	1	1	1	4.36	64	2	19	20.33	C	2
Felidae	Acinonyx jubatus[2]	cheetah	1.4	1	1.4	48.5	92.1	3.7	22	825	C	2

(Table S3 continued)

Family	Genus and species	Common Name	GS[1]	Cohesion	FGS	Mass[1]	Gest. Len.	WA	Longevity	HR[1]	Diet	Forelimb
Felidae	Puma concolor[2]	puma	1	1	1	51.75	91.54	2	23.8	525	C	2
Canidae	Alopex lagopus[4]	arctic fox	2	2	1	3.66	52.24	1.67	16.16	19.25	C	1
Canidae	Vulpes vulpes[4]	red fox	2	2	1	7	54.35	1.65	21.3	20.2	O	1
Canidae	Lycaon pictus[4]	African hunting dog	8	4	8	25	71.03	3	17	1020	C	1
Canidae	Canis mesomelas[4]	black-backed jackal	2	2	2	7.16	61.25	2.13	16.7	7.39	C	1
Canidae	Canis latrans[4]	coyote	4.33	2	2	11.45	62	2.06	21.83	44	C	1
Canidae	Canis lupus[4]	grey wolf	7	3	6.5	45	64.32	1.84	20.6	266 [6]	C	1
Ursidae	Melursus ursinus[4]	sloth bear	1	1	1	90	60	2.5	40	25.8	O	3
Ursidae	Ursus americanus[1]	black bear	1	1	1	118.75	60	5.5	34	28.46 [7]	O	2
Ursidae	Ursus maritimus[4]	polar bear	1	1	1	337.5	60	11.71	45	135000	C	2
Procyonidae	Procyon lotor[4]	raccoon	1	1	1	6.55	64.08	3.5	20.58	6.22	O	4
Procyonidae	Nasua nasua[4]	brown-nosed coati	20	3	1	4.6	73.5	4	23.7	5	O	3
Mustelidae	Taxidea taxus[4]	American badger	1	1	1	7.43	49	2	26	7.85	C	1
Mustelidae	Eira barbara[1]	tayra	1	1	1	4.85	65	3.33	22.3	12.33	O	3
Mustelidae	Gulo gulo[4]	wolverine	1	1	1	11.75	35	2.88	17.33	315.3	C	2
