# Supplementary material for: Multiple Determinants of Whole and Regional Brain Volume among Terrestrial Carnivorans
Source: PLoS One. 2012 Jun 13;7(6):e38447. doi: 10.1371/journal.pone.0038447 (PMC3374790; doi:10.1371/journal.pone.0038447)
Supplement: Table S4 — Model selection for best PGLS models, comparing models fitting a fixed brownian motion model (equivalent to using independent contrasts), a fixed ‘no effect of phylogeny’ model, and a model allowing lambda to take its MLE. (PDF) [file pone.0038447.s004.pdf]

Table S4

		AICc	df	dAICc	weight
Relative brain volume (skull)					
	Brownian motion	13.8	9	0	0.7875
	MLE	17.1	10	3.3	0.1507
	No phylogeny	18.9	9	5.1	0.0618
Relative brain volume (mass)					
	Brownian motion	16.9	9	0	0.86231
	MLE	20.7	10	3.8	0.13165
	No phylogeny	26.8	9	9.9	0.00604
Relative ACC					
	Brownian motion	82.4	9	0	0.75638
	MLE	84.7	10	2.3	0.24202
	No phylogeny	94.7	9	12.3	0.0016
Relative PCC					
	MLE	60.2	10	0	0.37
	No phylogeny	60.4	9	0.2	0.342
	Brownian motion	60.7	9	0.5	0.288
Relative Cerebellum					
	No phylogeny	19	9	0	0.391
	MLE	19.3	10	0.3	0.329
	Brownian motion	19.6	9	0.7	0.281
Relative Cerebrum					
	MLE	10.9	10	0	0.84793
	No phylogeny	14.4	9	3.5	0.14971
	Brownian motion	22.7	9	11.8	0.00236
